# Soluble NKG2DLs Are Elevated in Breast Cancer Patients and Associate with Disease Outcome

**DOI:** 10.3390/ijms25074126

**Published:** 2024-04-08

**Authors:** Anna Seller, Christian M. Tegeler, Jonas Mauermann, Tatjana Schreiber, Ilona Hagelstein, Kai Liebel, André Koch, Jonas S. Heitmann, Sarah M. Greiner, Clara Hayn, Dominik Dannehl, Tobias Engler, Andreas D. Hartkopf, Markus Hahn, Sara Y. Brucker, Helmut R. Salih, Melanie Märklin

**Affiliations:** 1 Clinical Collaboration Unit Translational Immunology, German Cancer Consortium (DKTK), Department of Internal Medicine, University Hospital Tübingen, Otfried-Müller-Str. 10, 72076 Tübingen, Germany; anna.seller@med.uni-tuebingen.de (A.S.);; 2 Department of Women’s Health, University Hospital Tübingen, Calwerstraße 7, 72076 Tübingen, Germany; 3 Cluster of Excellence iFIT (EXC2180) “Image-Guided and Functionally Instructed Tumor Therapies”, University of Tübingen, Röntgenweg 11, 72076 Tübingen, Germany; 4 Department of Peptide-Based Immunotherapy, Institute of Immunology, University Hospital Tübingen, Otfried-Müller-Str. 10, 72076 Tübingen, Germany

**Keywords:** NKG2DL, serum marker, breast cancer, DCIS, survival, MIC, ULBP

## Abstract

Ligands of the natural killer group 2D (NKG2DL) family are expressed on malignant cells and are usually absent from healthy tissues. Recognition of NKG2DLs such as MICA/B and ULBP1-3 by the activating immunoreceptor NKG2D, expressed by NK and cytotoxic T cells, stimulates anti-tumor immunity in breast cancer. Upregulation of membrane-bound NKG2DLs in breast cancer has been demonstrated by immunohistochemistry. Tumor cells release NKG2DLs via proteolytic cleavage as soluble (s)NKG2DLs, which allows for effective immune escape and is associated with poor prognosis. In this study, we collected serum from 140 breast cancer (BC) and 20 ductal carcinoma in situ (DCIS) patients at the time of initial diagnosis and 20 healthy volunteers (HVs). Serum levels of sNKG2DLs were quantified through the use of ELISA and correlated with clinical data. The analyzed sNKG2DLs were low to absent in HVs and significantly higher in BC patients. For some of the ligands analyzed, higher sNKG2DLs serum levels were associated with the classification of malignant tumor (TNM) stage and grading. Low sMICA serum levels were associated with significantly longer progression-free (PFS) and overall survival (OS). In conclusion, we provide the first insights into sNKG2DLs in BC patients and suggest their potential role in tumor immune escape in breast cancer. Furthermore, our observations suggest that serum sMICA levels may serve as a prognostic parameter in the patients analyzed in this study.

## 1. Introduction

Breast cancer (BC) is the most common cancer in women worldwide, and approximately 12.5% of women will develop BC during their lifetime [1,2]. The diagnosis of BC is still based on invasive methods, and non-invasive markers for diagnosis and risk stratification have not been clinically established.

BC is a heterogeneous disease and is clinically diagnosed through mammography, ultrasound, or magnetic resonance imaging (MRI), followed by tumor biopsy and histopathologic staining. Further assessment of clinical parameters is performed to determine the stage of disease, select the appropriate treatment regimen, and estimate the patient’s prognosis. Stratification according to the Union Internationale Contre le Cancer (UICC) or the classification of malignant tumors (TNM), which describes the size and extent of the primary tumor (T), spread to lymph nodes (N), and metastasis (M), or the grading (G) according to Elston and Ellis, which evaluates the differentiation of tumor cells through microscopy, are also used. The expression of hormone receptors (HRs), such as estrogen and progesterone or human epidermal growth factor receptor 2 (HER2), is used to classify patients and determine therapeutic options. Until now, soluble markers have not been considered in the diagnosis of breast cancer. Common and rather non-specific tumor markers such as lactate dehydrogenase (LDH), cardioembryonic antigen (CEA), and cancer antigen 15-3 (CA 15-3) are often measured at the time of diagnosis, which may be elevated in not only patients with various malignancies but also patients with liver disease or infections, but they do not have any impact on therapeutic decisions or prognostic value in breast cancer patients. Therefore, there is still a need for easily measurable parameters for assessment and risk stratification that can be determined in a non-invasive and simple manner. This highlights the importance of identifying biomarkers associated with breast cancer progression and prognosis.

Members of the killer group 2 member D ligand (NKG2DL) family are surface proteins that are primarily expressed on stressed, infected, or malignant cells and are low or absent on healthy cells, making them optimal tumor markers [3,4,5,6,7,8]. The NKG2DL family consists of MHC class I chain-related protein A/B (MICA/B) and UL16 binding protein 1-6 (ULBP1-6) molecules. NKG2DL surface expression on tumor cells has been shown to be associated with poor prognosis in cancer patients, including breast, ovarian, colorectal, and others [9,10,11,12,13,14,15]. In general, NKG2DLs are recognized by the activating immunoreceptor NKG2D on natural killer (NK) cells and cytotoxic T cells inducing cytotoxicity, thereby playing a critical role in anti-tumor immunity [16].

However, tumor cells are able to evade immune surveillance by shedding NKG2DLs from the cell surface, either through proteolytic cleavage or exosomal release of NKG2DLs [3,17,18,19,20,21,22]. This allows tumor cells to escape immune cell control and cytotoxic activity. Metalloproteases cleave all members of the NKG2DL family, thereby reducing membrane-bound ligands and increasing soluble NKG2DL (sNKG2DL) levels in the blood. In contrast, exosomal release is only possible for ligands such as ULBP1, ULBP3, or the MICA allele *008, which are bound to the membrane through a GPI anchor [23,24,25,26]. In addition, there are numerous findings from previous research regarding the role of secreted ligands in NK cell inhibition or NK cell activation; some studies show that free NKG2D receptors on immune cells are occupied by soluble ligands and consequently internalized in both NK and CD8 T cells, preventing recognition of other tumor cells [21,27,28]. Furthermore, the continuous activation of NKG2D has been shown to down-modulate the activity of other NK cell receptors [29,30]. However, there have also been important breakthroughs in the literature showing that secreted soluble ligands that bind to activating immune receptors do not always have an inhibitory effect, but, for example, in the case of the growth factor PD-L1, the ligand is able to inhibit the activity of NK cells, and in the case of the growth factor PDGF-DD to the NK cell receptor NKp44, it can also directly activate NK cells and thus induce the secretion of interferon gamma (IFN)-γ and tumor necrosis factor (TNF) by NK cells, which stop the growth of tumor cells [31,32]. Furthermore, Deng et al. showed that soluble ligands can also indirectly activate NK cell functions; in their mouse model, a secreted soluble form of MULT1, a high-affinity NKG2D ligand, was able to induce NK cell activation and tumor rejection. This was achieved in part by reversing a global desensitization of NK cells caused by the binding of NKG2D membrane ligands to tumor-associated cells, such as myeloid cells [33]. This suggests that sensitive NK cell activation depends on a balance of signaling through activating and inhibitory receptors.

For more than 20 years, the role of sNKG2DLs, especially sMICA, has been investigated in various tumor diseases, especially in leukemia [9,21,22,33]. It has been shown that sNKG2DLs in patient serum reduce NKG2D expression on NK cells, resulting in impaired anti-tumor reactivity [34]. While the membrane-bound expression of NKG2DLs on tumor cells and their interaction with NK cells or cytotoxic T cells has been studied, data on sNKG2DLs in breast cancer have been rather neglected. To date, the potential role of sNKG2DLs in breast cancer pathogenesis, therapy, and prognosis is poorly understood. Elevated serum levels of sMICA/B and sULBP2 have been associated with adverse clinical outcomes and metastasis in various cancers, including breast, colorectal, ovarian, prostate, lung, and other malignancies [9,10,11,12,13,14,15].

Here, we investigate the serum levels of sMICA, sMICB and sULBP1-3 in 140 breast cancer (BC) patients and 20 ductal carcinoma in situ (DCIS) patients compared to 20 healthy volunteers (HVs) and their association with clinical parameters and survival.

## 2. Results

### 2.1. Clinical Characteristics of the Patient Population Studied

In this study, we analyzed serum samples from 140 BC and 20 DCIS patients at the time of diagnosis and before the initiation of any therapy. The median age of the patients at the time of sample collection was 56 years (range 26–92). The vast majority (98%) had no special cancer subtype (NST) and only 2% had an invasive lobular carcinoma (ILC) subtype. In terms of tumor size, 19% had small T1 tumors, 40% had T2 stage tumors, 24% had T3 stage tumors, and 18% had large T4 stage tumors. The distribution of lymph node status was well balanced with 48% lymph node-negative and 52% lymph node-positive patients. Most of the patients had no distant metastases at the time of sampling (86% vs. 14%) and were in UICC stage II (42%) or III (29%), while only 15% were in UICC stage I and 14% were in UICC stage IV stage. Regarding grading, 4%, 46%, and 51% of the tumors were G1, G2, and G3 grade carcinomas, respectively. Regarding receptor status, HR-positive/HER2-negative (HR+/HER2−) tumors were predominant (54%), followed by the triple-negative breast cancer (TNBC) group (24%). Only 13% were HR+/HER2+ and 9% were HR-/HER2+. Common but non-specific tumor markers such as CEA, CA 15-3, and LDH were assessed through blood analysis at the time of diagnosis; on average, the levels were found to be below the reference range (Table 1).

### 2.2. Distribution of sNKG2DL Serum Levels

**Figure 1:**
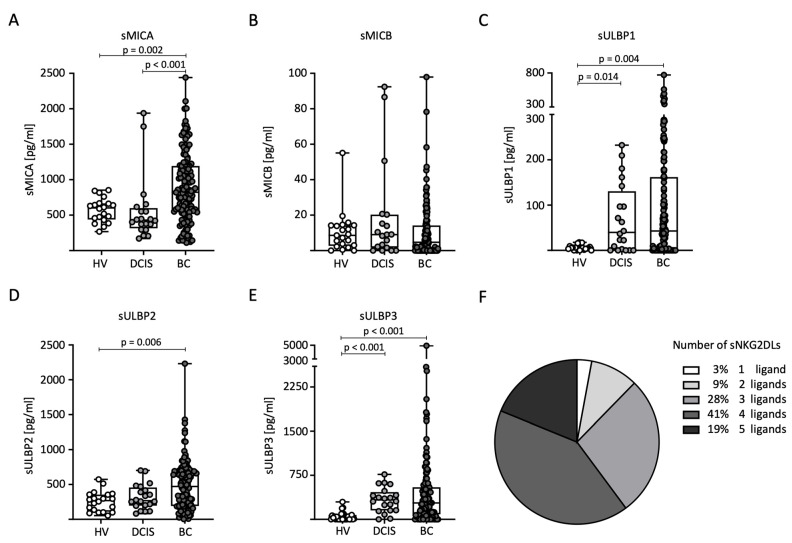
Distribution of sNKG2DL serum levels in HVs, DCIS patients, and BC patients. (**A**–**E**) Serum levels of sMICA (**A**), sMICB (**B**), sULBP1 (**C**), sULBP2 (**D**), and sULBP3 (**E**) were determined through the use of ELISA in the HVs (*n* = 20), DCIS patients (*n* = 20), and BC (*n* = 140) patients. (**F**) Frequency distribution of BC patients with one or more sNKG2DLs among all sNKG2DL-positive BC patients. sNKG2DL positivity was defined by serum levels above the first quartile.

First, we compared sNKG2DL serum levels between the HVs (*n* = 20), DCIS patients (*n* = 20), and BC (*n* = 140) patients. Notably, the serum levels of all sNKG2DLs tested, except sMICB, were significantly elevated in BC patients compared to HVs (Figure 1A–E). In addition, higher levels of sMICA were detected in BC patients compared to DCIS patients (Figure 1A). For sULBP1 and sULBP3, higher levels were detected in DCIS patients compared to HVs (Figure 1C,E). The majority of breast cancer patients (88%) had sNKG2DL serum levels above the first quartile for three or more NKG2DLs (Figure 1F). Because only 12 breast cancer patients had elevated MICB serum levels above the ELISA detection limit, further correlation between MICB and the clinical parameters was not performed.

### 2.3. sNKG2DL Serum Levels Correlate with TNM Stage

**Figure 2:**
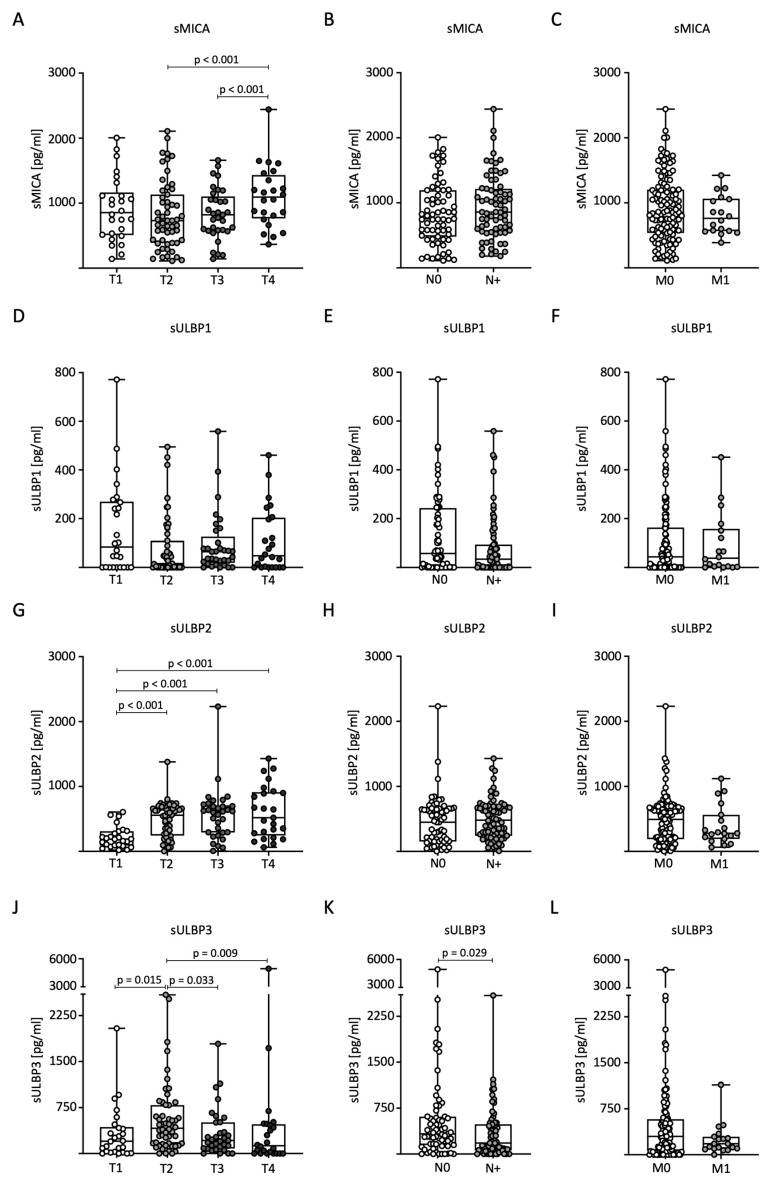
Distribution of sNKG2DL serum levels in BC patients according to TNM stage. (**A**–**L**) Serum levels in BC patients (*n* = 140) were determined through the use of ELISA and distributed according to tumor size (T stage), lymph node status (N stage), and distant metastasis (M stage). sMICA (**A**–**C**), sULBP1 (**D**–**F**), sULBP2 (**G**–**I**), and sULBP3 (**J**–**L**).

Next, we analyzed sNKG2DL levels in relation to TNM classification in BC patients. For sMICA, significantly higher serum levels were measured in T4 tumors compared to the T2 and T3 stages (Figure 2A). No significant differences were observed for nodal status (N) and distant metastasis (M) (Figure 2B,C). Serum levels of sULBP1 showed no significant differences with respect to TNM stage (Figure 2D–F). For sULBP2, significantly higher serum levels were measured in tumor stages T2, T3, and T4 compared to stage T1 (Figure 2G). As with sMICA, there were no significant differences in nodal status or distant metastases (Figure 2H,I). Serum levels of sULBP3 were significantly higher in patients with stage T2 tumors than in the other stages (Figure 2J). Furthermore, higher levels were observed in lymph node-negative patients (Figure 2K). There were no significant differences in the occurrence of distant metastases (Figure 2L).

### 2.4. Range of sNKG2DL Serum Levels According to Tumor Grading and Receptor Status

**Figure 3:**
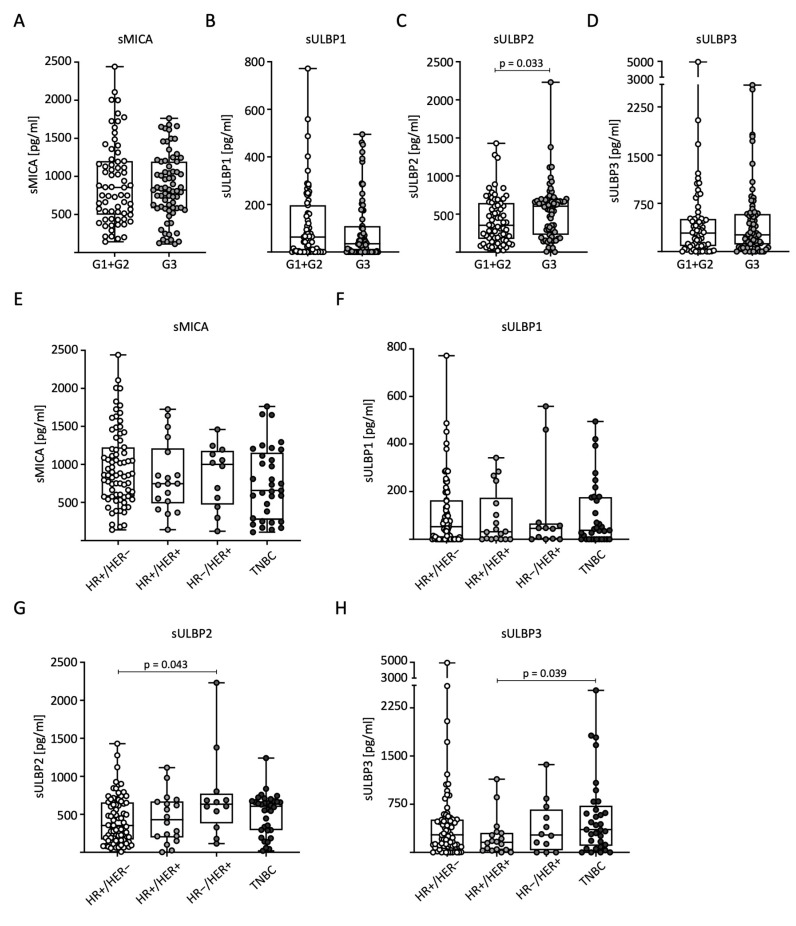
Distribution of sNKG2DL serum levels in BC patients according to tumor grading, hormone, and growth receptor status. Serum sNKG2DL levels were determined through the use of ELISA in BC patients (*n* = 140). (**A**–**D**) Patients were divided into low-grade (G1 + G2) and high-grade (G3) tumors. (**E**–**H**) Patients were divided into subtypes according to HR and HER2 receptor status. HR, hormone receptor; HER2, HER2 receptor; TNBC, triple-negative breast cancer.

To analyze sNKG2DL distribution in relation to tumor grading (G) according to Elston and Ellis, we divided the patients into low-grade (combined G1 + G2, *n* = 69) and high-grade (G3, *n* = 71) tumors for further analysis. Significantly higher serum levels of sULBP2 were observed in G3 than in G1 + G2 (Figure 3C). No significant differences were observed for the other sNKG2DLs (Figure 3A–D). We also examined the correlation between sNKG2DL serum levels, HR status, and HER2 expression. The BC patients were divided into four subtypes: HR+/HER2−, HR+/HER2+, HR−/HER2+, and TNBC. In conclusion, the measured serum levels in general and for sMICA in particular were relatively homogeneously distributed across the different receptor subtypes. For sMICA and sULBP1, no significant difference between the subtypes was observed (Figure 3E,F). For sULBP2, significantly higher serum levels were observed in HR-/HER2+ than in HR+/HER2- tumors (Figure 3G), and for sULBP3, significantly higher serum levels were observed in TNBC than in HR+/HER2+ tumors (Figure 3H).

### 2.5. Impact of sNKG2DLs on Progression-Free Survival (PFS) and Overall Survival (OS)

**Figure 4:**
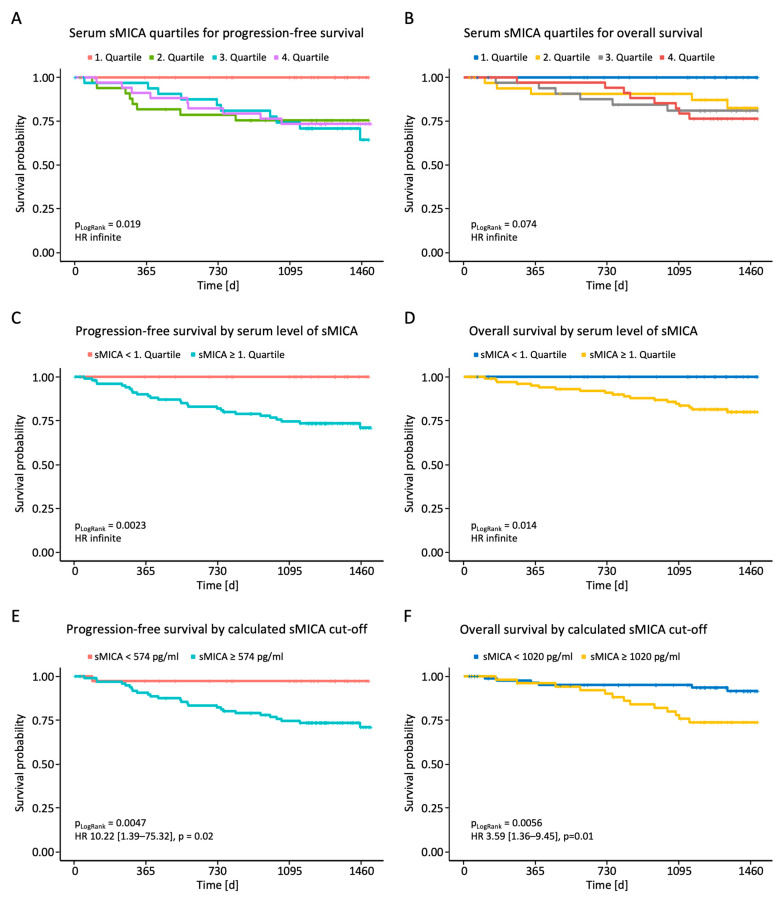
Correlation between sMICA serum levels and PFS and OS in BC patients. Serum levels of sMICA were determined through the use of ELISA in breast cancer patients (*n* = 140) and correlated with PFS and OS. (**A**,**B**) Correlation between sMICA and PFS and OS in BC patients according to quartiles. (**C**,**D**) Correlation between sMICA and PFS and OS in BC patients below and above the 1st quartile. (**E**,**F**) Correlation between sMICA and PFS and OS in BC patients according to the calculated cut-off values. PFS, progression-free survival; OS, overall survival; %; percent; *p*, *p*-value.

Finally, we analyzed whether the measured sNKG2DL serum levels correlated with PFS or OS. Initial analyses dividing the breast cancer patients into the four quartiles of measured sMICA serum levels showed superior PFS and a trend toward longer OS for the first quartile (Figure 4A,B). After dividing the patients into two groups with serum levels below or above the first quartile, we observed a significantly shortened PFS and OS in breast cancer patients with high sMICA serum levels (Figure 4C,D). No effect of sULBP1-3 serum levels on PFS and OS was observed (Supplementary Figure S1). To further consolidate the observed effect of sMICA levels on survival, receiver operating characteristics (ROC) analysis was performed using serum sMICA levels and the value of the highest Youden index as a cut-off (574 pg/mL for PFS; 1020 pg/mL for OS). The calculated sMICA cut-off allowed for further separation of cases with better or worse prognosis, as shown in the Kaplan–Meier analysis (Figure 4E,F), and confirmed the above findings: patients with high serum sMICA levels had significantly shorter PFS and OS.

### 2.6. Analysis of NK Cell Function and NKG2D Receptor Expression

**Figure 5:**
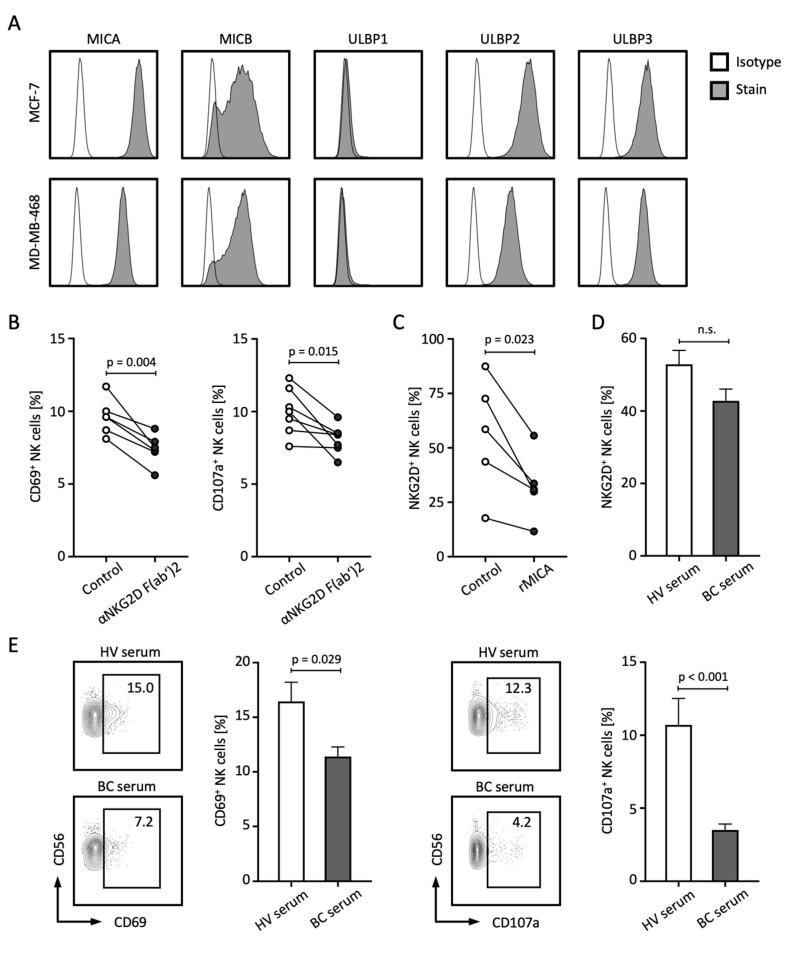
sNLG2DLs impair NK cell function through the downregulation of NKG2D. (**A**) Surface expression of the indicated NKG2DL was stained with the respective antibody and isotype control and assessed through the use of flow cytometry. (**B**) PBMCs from HVs (*n* = 4) were co-cultured with MCF-7 or MDA-MB-468 tumor cells (E:T 4:1) in the presence or absence of anti-NKG2D F(ab’)2 fragments for 4 h and NK cell activation and degranulation was analyzed by CD69 and CD107a expression using flow cytometry, respectively. (**C**) PBMCs from HVs (*n* = 5) were co-cultured with soluble rMICA for 48 h and NKG2D expression on NK cells was assessed through the use of flow cytometry. (**D**) PBMCs from the HV (*n* = 6) were co-cultured with serum from the HVs (*n* = 9) or BC patients with high sNKG2DL levels (*n* = 6) for 24 h and NKG2D expression on NK cells was assessed through the use of flow cytometry. (**E**) PBMCs from the HVs (*n* = 6) were co-cultured with MCF-7 or MDA-MB-468 tumor cells (E:T 4:1) with serum from the HVs (*n* = 9) or BC patients with high sNKG2DL levels (*n* = 6) for 4 h and NK cell activation and degranulation was analyzed by CD69 and CD107a expression using flow cytometry, respectively. HVs, healthy volunteers; BC, breast cancer; E:T, effector-to-target ratio; %; percent; *p*, *p*-value; n.s., not significant.

The release of NKG2DL not only contributes to the failed immune recognition of tumor cells by NK and T cells, but sNKG2DLs are also capable of mediating tumor-promoting functions themselves [8,21]. In the following, we investigated which other mechanisms might lead to the poorer prognosis of BC patients with elevated sNKG2DL levels. First, we analyzed the membrane-bound expression of NKG2DL in the BC cell lines MCF-7 and MDA-MB-468 and observed relevant expression of MICA, MICB, ULBP2, and ULBP3, while ULBP1 was not expressed (Figure 5A). To analyze the role of NKG2DL–NKG2D interaction in the immune surveillance of BC by NK cells, we co-cultured peripheral blood mononuclear cells (PBMCs) with BC cell lines in the presence or absence of an NKG2D blocking antibody. Blockade of NKG2D resulted in decreased activation and degranulation of NK cells as analyzed by CD69 and CD107a expression, respectively (Figure 5B). Treatment of PBMCs with soluble recombinant MICA (rMICA) resulted in a significant decrease in NKG2D expression on NK cells (Figure 5C). Similar trends were observed when PBMCs were treated with serum from BC patients with high levels of sNKG2DL compared to HV serum (Figure 5D). Of note, comparable effects of NKG2D expression and downregulation were observed with T cells (Supplementary Figure S2). Finally, co-culture experiments with PBMCs and BC cell lines in the presence of serum from HVs or BC patients showed that the presence of BC serum led to significantly reduced expression of CD69 and CD107a on NK cells (Figure 5E).

In conclusion, in this study, we were able to show that the analyzed sNKG2DLs serum levels of sMICA and sULBP1-3 were significantly higher in BC patients, whereas the measured sNKG2DLs serum levels were low to absent in the HVs. In particular, we were able to show that low sMICA serum levels were associated with significantly longer PFS and OS. Furthermore, we were able to show that sMICA leads to the downregulation of NKG2D and that the serum of BC patients restricts NK cell activity.

## 3. Discussion

Stress-induced NKG2DLs are expressed on the surface of various cancers but are largely absent on healthy cells, suggesting that the NKG2D receptor plays an important role in immune surveillance and making NKG2D one of the most intensively studied immune receptors in the last decade [3,16,35,36]. The expression and function of NKG2DL have been extensively studied in various cancers, and high expression of membrane-bound NKG2DLs have been shown to be associated with poor prognosis [3,4,9,10,11,14,15,21,24,37,38,39,40]. Given this, and the fact that tumor cells employ efficient strategies to evade the membrane-bound NKG2DL-mediated anti-tumor reactivity of NK cells, it is not surprising that tumor cells can shed NKG2DLs from their cell surface, resulting in reduced expression levels and thus the amount of stimulatory signaling that determines whether or not NK cell responses are elicited [8]. The role of sNKG2DLs in BC patients is understudied, as the focus has largely been on membrane-bound NKG2DL. Studies by Zhang et al., Dhar et al., Bauer et al. and others have demonstrated opposing regulatory effects of membrane-bound and sNKG2DLs on tumor immunity [3,16,41]. NKG2D receptor signaling depends on adaptor molecules to initiate cell activation. In particular, membrane-bound NKG2DLs stimulate anti-tumor immunity through cross-linking and conformational changes in the NKG2D receptor, whereas sNKG2DLs suppress anti-tumor immunity through several mechanisms. These mechanisms include downregulation of NKG2D expression on effector cells, leading to T cell and NK cell dysfunction, and impaired NK cell self-renewal in tumor hosts, disrupting NK cell homeostasis [3,16,41]. Our results regarding NKG2D–NKG2DL interaction in breast cancer immune surveillance underscore the inhibitory effects of sNKG2DLs on NKG2D-mediated NK cell activation. This supports already existing data showing that tumor-derived sNKG2DLs in the sera of cancer patients can cause systemic NKG2D downregulation, further compromising NK cell immune surveillance [3,18]. Consequently, the presence of sNKG2DLs in patient sera may contribute to a worse prognosis in breast cancer patients.

Since previous studies have shown that during malignant transformation, cells not only express NKG2DLs but also increasingly cleave and thereby release them in order to evade the immune system, it can be assumed that not only the membrane-bound ligands are increased but also the soluble ligands in the serum [17,19,33,42]. Roshani et al. observed higher sMICA levels in 49 BC patients compared to healthy subjects, as well as an inverse correlation between sMICA serum levels and NKG2D on NK cells, supporting the aforementioned immune escape mechanisms of breast cancer cells [43]. Our results confirmed this hypothesis: higher serum levels of sMICA, sULBP1, sULBP2, and sULBP3 were measured in breast cancer patients compared to healthy subjects. sMICB levels appeared to be rather low in breast cancer patients, which could either indicate that the cleavage of MICB is less relevant in breast cancer or that the breast tumors of the analyzed patients did not initially express membrane-bound MICB at relevant levels. The increased sULBP1 and sULBP3 levels in DCIS patients compared to healthy volunteers may indicate possible overexpression of ULBP1 and ULPB3 already on DCIS tissue and thus ligand cleavage as an early mechanism of immune evasion. However, this could not be demonstrated in our cohort, but may be of interest for further evaluation as NKG2DL expression and function has not yet been studied in DCIS. Except for the increased sULBP1 and sULBP3 serum levels in DCIS and BC patients compared to HVs, we could not identify clear patterns for association with grading or growth or hormone receptors. Even if there were slightly elevated sULBP3 levels in the TNBC samples and in T2 tumors, we do not consider this to be of prognostic relevance. This is supported by the data on PFS and OS data for sULBP1 and sULBP3, which also showed no significant difference. For sULBP2, a clear correlation between tumor staging and grading was observed, suggesting increased cleavage of ULBP2 in T4 tumors or in less differentiated G3 tumors. Slightly elevated serum sULBP2 levels were also observed in HR−/HER+ samples, but in the same line of argument as for ULBP1 and ULBP3, we do not believe that this would have a prognostic impact. In general, our data do not allow a clear conclusion regarding the relationship between hormone/HER2 receptor status and the measured sNKG2DL serum levels. Further studies are needed to make more definitive statements.

Our results obtained for sMICA proved to be the most reliable and promising for further clinical evaluation. Low serum sMICA levels were associated with longer PFS and OS. This may suggest that sMICA may serve as a prognostic marker to assess clinical progression in breast cancer. Our data are consistent with the findings of de Kruijf et al. that high expression levels of membrane-bound MICA/B and ULBP2 resulted in prolonged progression-free survival because immune surveillance by the NKG2D–NKG2DL axis was not impaired [6]. They support the hypothesis that the cleavage of NKG2DLs—and thus lower expression of membrane-bound ligands but higher levels of soluble ligands—could systemically downregulate NKG2D receptor expression and result in the impaired anti-tumor reactivity of NK and T cells, leading to poorer survival. These results can be supported by our data and further by Schmiedel et al. and others [4,7,18,40,44,45]. The diagnostic potential of sNKG2DLs, especially sMICA, as a serum marker appears to be valuable, but critically, it must be kept in mind that the release of sNKG2DLs is not specific to breast cancer but has been shown to be present in leukemia, cervical, ovarian, prostate, gastric, melanoma, and other cancers [3,10,11,14,15,24,37,38,39].

To date, several immunotherapeutic approaches have been proposed to target the NKG2D–NKG2DL axis for cancer therapy [46]. One promising therapeutic option is the use of small molecule-shedding inhibitors to prevent immune escape [47,48,49]. Others suggest the promising use of antibodies, either to reduce serum levels of soluble ligands or to prevent shedding as well [50,51]. In March 2024, the anti-MICA/B antibody CLN-619, which inhibits shedding, received FDA approval for the treatment of relapsed/refractory multiple myeloma [52]. In 2019, Paczulla et al. demonstrated that acute myeloid leukemia (AML) patients whose AML cells do not express NKG2DLs have effective immune evasion mechanisms. These are often responsible for disease relapse despite chemotherapy [53]. The study also showed that Poly-ADP-ribose polymerase 1 (PARP1) repressed NKG2DL expression, and treatment with PARP inhibitors successfully (re-)induced NKG2DL expression. Translating these findings to BC, there is an obvious need for a prospective study to analyze sNKG2DLs before and after PARP inhibitor therapy in BRCA mutated patients. Maccalli et al. described that high levels of sNKG2DLs in melanoma patients before therapy correlated with reduced survival in response to immune checkpoint blockade therapy, suggesting that serum levels of sNKG2DLs may be used as a serum marker to select melanoma patients for immune checkpoint therapy [37]. Finally, Alcazar et al. investigated the response and resistance mechanisms of immune checkpoint therapy in a rat model of breast cancer [54]. Their results suggest that the downregulation of NKG2D by sNKG2DLs may be a mechanism for immune escape in a subset of luminal tumors and that upregulation of PD-L1 may not provide additional benefit, explaining the lack of response to the immune checkpoint inhibitor pembrolizumab. This should also be investigated in a larger BC cohort. In addition, it would be interesting to further analyze whether sNKG2DLs are useful for assessing the response to neoadjuvant therapy in breast cancer patients. To date, response assessment to neoadjuvant therapy in breast cancer is primarily performed using imaging modalities such as MRI, ultrasound, and mammography [55,56,57,58,59]; therefore, sNKG2DL levels in relation to neoadjuvant therapy may serve as a complementary, easy-to-use, cost-effective, and non-invasive tool for response assessment that could benefit many patients.

In conclusion, the results of this study not only advance our understanding of the biological significance of sNKG2DLs in BC but also suggest that specific sNKG2DLs such as sMICA could potentially serve as a prognostic indicator for evaluating clinical outcomes in BC. Furthermore, these findings suggest that sNKG2DLs may play a critical role in shaping future immunotherapeutic strategies by either suppressing or enhancing their interactions with the immune system.

Overall, we anticipate that this study will provide novel insights into the involvement of sNKG2DLs in BC and lay the groundwork for further investigation in this area.

## 4. Materials and Methods

### 4.1. Patients

Between 2016 and 2020, blood samples were collected from patients with primary breast cancer and DCIS patients at the Breast Center of the Department of Women’s Health at the University Hospital of Tübingen, Germany, at the time of diagnosis and before the initiation of any therapy. During the follow-up period, all patients received standardized treatment according to national guidelines, including systemic therapy, surgery, and radiation. Serum samples from HVs were collected at the University Hospital Tübingen, Germany. Serum was separated via centrifugation at 3220× *g* for 10 min and the supernatant was stored at −80 °C. Informed consent was obtained in accordance with the guidelines of the Declaration of Helsinki. The study was approved by and conducted in accordance with the policies of the local ethics committees (reference number 13/2007V). The diagnosis was confirmed through biopsy and histopathologic findings. Clinical data on patient and tumor characteristics were collected from the original medical records and pathology reports. Survival data were collected during follow-up up to 1500 days after surgery. Table 1 summarizes the characteristics of the breast cancer patients.

### 4.2. PBMCs and Cell Lines

PBMCs were isolated from healthy donors through density gradient centrifugation using Pancoll Cell Separation Solution (PAN-biotech, Aidenbach, Germany). Human breast cancer cell lines MCF-7 and MDA-MB-468 were obtained from the DSMZ. All cell lines were cultured in Dulbecco’s modified Eagle medium (Thermo Fisher Scientific, Waltham, MA, USA) supplemented with 10% heat-inactivated fetal calf serum (PAN-biotech, Aidenbach, Germany), 100 U/mL penicillin (Merck, Darmstadt, Germany), and 100 μg/mL streptomycin (Merck, Darmstadt, Germany) at 37 °C with 5% CO_2_. All cell lines were routinely tested for mycoplasma contamination.

### 4.3. Flow Cytometry Analysis

For specific staining of membrane-bound NKG2DL, human MICA, MICB, ULBP1, ULBP2, and ULBP3 antibodies were used as described previously [34,60]. Briefly, after blocking of the cells with human IgG (Sigma Aldrich, St. Louis, MO, USA), the cells were stained with the respective primary antibodies (Abs) and isotype controls (10 µg/mL each) followed by goat anti-mouse PE conjugate (Dako, Glostrup, Denmark) as a secondary reagent. NK cell activation and degranulation were assessed by culturing PBMCs from healthy donors with BC cell lines at an effector-to-target (E:T) ratio of 4:1 for 4 h in the presence or absence of 20% serum from healthy donors or BC patients, followed by flow cytometric staining. NK cells were identified using CD56-PeCy7 (clone: HCD56), TCR-APC/Fire750 (clone: B1), CD69-FITC (clone: FN50), CD107a-BV421 (clone: H4A3) (all BioLegend, San Diego, CA, USA), NKG2D-PE (clone: 1D11) (R&D Systems, Minneapolis, MN, USA), and 7-AAD (BioLegend, San Diego, CA, USA). For NKG2D receptor blockade, F(ab’)2 fragments of an anti-NKG2D mAb (clone 6H7 kindly provided by Amgen Inc., Thousand Oaks, CA, USA) were added to the co-culture as described previously [53]. For downmodulation of NKG2D, PBMCs were cultured with rMICA or human serum. Measurements were performed using FACS Fortessa (BD Biosciences, Heidelberg, Germany), and the data were analyzed using FlowJo-V10 software (BD Biosciences, Heidelberg, Germany).

### 4.4. Enzyme-Linked Immunosorbent Assay (ELISA)

Serum levels of sMICA, sMICB, and sULBP1-3 were determined through the use of sandwich ELISA, as previously described [33,34]. Briefly, plates were coated with the capture mAb at 2 μg/mL in phosphate-buffered saline (PBS), then blocked by the addition of 100 μL 10% non-fat dry milk in PBS for 2 h at 37 °C, and washed. Sera (after 1:2 dilution in 5% non-fat dry milk in PBS) were added in triplicate, and the plates were incubated for 2 h at 37 °C. Recombinant MICA, MICB, ULBP1, ULBP2, and ULBP3 were purchased from R&D Systems. After incubation, the plates were washed, and the detection mAb was added at 1 μg/mL 5% non-fat dry milk for 1.5 h at 37 °C. After washing, anti-mouse IgG2a-HRP was added to detect sMICB, sULBP1, and sULBP2, and anti-mouse IgG1-HRP was added to detect sULBP3 for 45 min at 37 °C (both from SouthernBiotech, Birmingham, AL, USA; 1:5000 in 2.5% non-fat dry milk in PBS). The plates were then washed and developed using the TMB peroxidase substrate system (KPL, sera care). The following Abs were used for the detection of sMICB, sULBP1, sULBP2, and sULBP3: coating, anti-MICB BAMO1 (IE9), anti-ULBP1 AUMO5, anti-ULPB2 BUMO1 (1D11), and anti-ULBP3 AF1517 (R&D Systems); detection, anti-MICB BMO2 (IH1), anti-ULBP1 AUMO2, anti-ULBP2, and anti-ULBP3 CUMO3. For the detection of MICA, a commercial kit (R&D Systems) was used according to the manufacturer’s instructions. All concentrations reported are the mean of triplicates.

### 4.5. Software and Statistical Analysis

Data are presented as the median with the range, box plots with the median and 25th and 75th quartiles, min/max whiskers, and individual data points or the mean ± standard error of the mean (SEM). Continuous data were tested for distribution, and individual groups were tested using an unpaired Mann–Whitney U test or Kruskal–Wallis test or paired Student’s *t* test. Missing data were included in tables and descriptive analyses. The distribution of overall survival (OS) and progression-free survival (PFS) was calculated using the Kaplan–Meier method. The log-rank test was used to test for differences in survival between groups. To determine the predictive cut-off value, we first divided the breast cancer patients into the four quartiles of measured serum levels. After this step, a low and a high group was created according to serum levels below or above the first quartile. Receiver operating characteristic (ROC) analysis was performed using JMP Pro software (SAS Institute, Cary, NC, USA, v.16) and the value of the highest Youden index was used as the cut-off for further analysis. The cut-off values allowed for further separation of cases with better or worse prognosis, as shown in the Kaplan–Meier analysis, for example. Statistical analyses were performed with JMP Pro. *p*-values < 0.05 were considered significant. Graphs were generated using GraphPad Prism v.9.1.2 and R version 4.3.1 [61,62].

## Figures and Tables

**Table 1:** Clinical characteristics of the breast cancer patients. *n*, number of donors; %, percentage of patients; SD, standard deviation; TNM, classification of malignant tumors; UICC; stages according to Union Internationale Contre le Cancer; grading, G; HR, hormone receptor; HER2, human epidermal growth factor receptor 2; TNBC, triple-negative breast cancer; LDH, lactate dehydrogenase; CEA, cardioembryonic antigen; CA 15-3, cancer antigen 15-3.

Patient Characteristics (*n* = 140)	
Age (in years, median [range])	56 (26–92)
**Breast cancer subtype (*n* [%])**	
NST (no special type)	137 (98)
ILC (invasive lobular carcinoma)	3 (2)
**TNM stage**	
**Tumor size (T) (*n* [%])**	
T1	26 (19)
T2	56 (40)
T3	33 (24)
T4	25 (18)
**Nodal status (N) (*n* [%])**	
N0	67 (48)
N+	73 (52)
**Distant metastases (M) (*n* [%])**	
M0	121 (86)
M1	19 (14)
**UICC stages (*n* [%])**	
UICC I	21 (15)
UICC II	59 (42)
UICC III	41 (29)
UICC IV	19 (14)
**Grading (acc. to Elston and Ellis) (*n* [%])**	
G1	5 (4)
G2	64 (46)
G3	71 (51)
**Receptor status (*n* [%])**	
HR+/HER2−	76 (54)
HR+/HER2+	18 (13)
HR−/HER2+	12 (9)
TNBC	34 (24)
**Serum marker levels (mean (±SD))**	
LDH (U/L, max. 250)	222.3 (±54)
CEA (µg/L, max. 5)	4.3 (±19.4)
CA 15-3 (kU/L, max. 33)	22.4 (±28.8)

## Data Availability

The data presented in this study are available from the corresponding author upon reasonable request. The data are not publicly available to protect sensitive patient information.

## Supplementary Materials

The following supporting information can be downloaded at: https://www.mdpi.com/article/10.3390/ijms25074126/s1.

## References

[1] Zentrum für Krebsregisterdaten R. (2023). Krebsdaten, Brustkrebs. https://www.krebsdaten.de/Krebs/DE/Content/Krebsarten/Brustkrebs/brustkrebs_node.html.

[2] (2023). American Cancer Society, Cancer Facts and Figures. https://www.cancer.org/cancer/types/breast-cancer/about/how-common-is-breast-cancer.html.

[3] Dhar P., Wu J.D. (2018). NKG2D and its ligands in cancer. Curr. Opin. Immunol..

[4] Zhang Y., Han C., Shao E., Sun L., Liu D. (2021). Expression, Prognosis, and Regulation of ULBP1, ULBP2, and ULBP3 in Human Breast Cancer. Preprint.

[5] Agaugue S., Hargreaves A., De Sousa P., De Waele P., Gilham D. (2018). 1179P—The high expression of NKG2D ligands on tumor and the lack of surface expression on healthy tissues provides a strong rationale to support NKG2D-based therapeutic approaches for cancer. Ann. Oncol..

[6] De Kruijf E.M., Sajet A., van Nes J.G., Putter H., Smit V.T., Eagle R.A., Jafferji I., Trowsdale J., Liefers G.J., van de Velde C.J. (2012). NKG2D ligand tumor expression and association with clinical outcome in early breast cancer patients: An observational study. BMC Cancer.

[7] Madjd Z., Spendlove I., Moss R., Bevin S., Pinder S.E., Watson N.F.S., Ellis I., Durrant L.G. (2007). Upregulation of MICA on high-grade invasive operable breast carcinoma. Cancer Immun..

[8] Salih H.R., Holdenrieder S., Steinle A. (2008). Soluble NKG2D ligands: Prevalence, release, and functional impact. Front. Biosci..

[9] Holdenrieder S., Stieber P., Peterfi A., Nagel D., Steinle A., Salih H.R. (2006). Soluble MICB in malignant diseases: Analysis of diagnostic significance and correlation with soluble MICA. Cancer Immunol. Immunother..

[10] Tamaki S., Kawakami M., Ishitani A., Kawashima W., Kasuda S., Yamanaka Y., Shimomura H., Imai Y., Nakagawa Y., Hatake K. (2010). Soluble MICB serum levels correlate with disease stage and survival rate in patients with oral squamous cell carcinoma. Anticancer. Res..

[11] Wu J.D., Higgins L.M., Steinle A., Cosman D., Haugk K., Plymate S.R. (2004). Prevalent expression of the immunostimulatory MHC class I chain-related molecule is counteracted by shedding in prostate cancer. J. Clin. Investig..

[12] Zhao Y.K., Jia C.M., Yuan G.J., Liu W., Qiu Y., Zhu Q.G. (2015). Expression and clinical value of the soluble major histocompatibility complex class I-related chain A molecule in the serum of patients with renal tumors. Genet. Mol. Res..

[13] Kshersagar J., Damle M.N., Bedge P., Jagdale R., Tardalkar K., Jadhav D., Jagadale S., Toro Y., Sharma R., Joshi M.G. (2022). Downregulation of MICA/B tumor surface expressions and augmented soluble MICA serum levels correlate with disease stage in breast cancer. Breast Dis..

[14] Paschen A., Sucker A., Hill B., Moll I., Zapatka M., Nguyen X.D., Sim G.C., Gutmann I., Hassel J., Becker J.C. (2009). Differential clinical significance of individual NKG2D ligands in melanoma: Soluble ULBP2 as an indicator of poor prognosis superior to S100B. Clin. Cancer Res..

[15] Rebmann V., Schütt P., Brandhorst D., Opalka B., Moritz T., Nowrousian M.R., Grosse-Wilde H. (2007). Soluble MICA as an independent prognostic factor for the overall survival and progression-free survival of multiple myeloma patients. Clin. Immunol..

[16] Bauer S., Groh V., Wu J., Steinle A., Phillips J.H., Lanier L.L., Spies T. (1999). Activation of NK Cells and T Cells by NKG2D, a Receptor for Stress-Inducible MICA. Science.

[17] Baragaño Raneros A., Suarez-Álvarez B., López-Larrea C. (2014). Secretory pathways generating immunosuppressive NKG2D ligands: New targets for therapeutic intervention. Oncoimmunology.

[18] Schmiedel D., Mandelboim O. (2018). NKG2D Ligands-Critical Targets for Cancer Immune Escape and Therapy. Front. Immunol..

[19] Salih H.R., Goehlsdorf D., Steinle A. (2006). Release of MICB molecules by tumor cells: Mechanism and soluble MICB in sera of cancer patients. Hum. Immunol..

[20] Mamessier E., Sylvain A., Bertucci F., Castellano R., Finetti P., Houvenaeghel G., Charaffe-Jaufret E., Birnbaum D., Moretta A., Olive D. (2011). Human breast tumor cells induce self-tolerance mechanisms to avoid NKG2D-mediated and DNAM-mediated NK cell recognition. Cancer Res..

[21] Groh V., Wu J., Yee C., Spies T. (2002). Tumour-derived soluble MIC ligands impair expression of NKG2D and T-cell activation. Nature.

[22] Salih H.R., Rammensee H.G., Steinle A. (2002). Cutting edge: Down-regulation of MICA on human tumors by proteolytic shedding. J. Immunol..

[23] Ashiru O., Boutet P., Fernández-Messina L., Agüera-González S., Skepper J.N., Valés-Gómez M., Reyburn H.T. (2010). Natural killer cell cytotoxicity is suppressed by exposure to the human NKG2D ligand MICA*008 that is shed by tumor cells in exosomes. Cancer Res..

[24] Raffaghello L., Prigione I., Airoldi I., Camoriano M., Levreri I., Gambini C., Pende D., Steinle A., Ferrone S., Pistoia V. (2004). Downregulation and/or release of NKG2D ligands as immune evasion strategy of human neuroblastoma. Neoplasia.

[25] Sheppard S., Ferry A., Guedes J., Guerra N. (2018). The Paradoxical Role of NKG2D in Cancer Immunity. Front. Immunol..

[26] Wiemann K., Mittrucker H.-W., Feger U., Welte S.A., Yokoyama W.M., Spies T., Rammensee H.-G., Steinle A. (2005). Systemic NKG2D down-regulation impairs NK and CD8 T cell responses in vivo. J. Immunol..

[27] Lundholm M., Schröder M., Nagaeva O., Baranov V., Widmark A., Mincheva-Nilsson L., Wikström P. (2014). Prostate tumor-derived exosomes down-regulate NKG2D expression on natural killer cells and CD8+ T cells: Mechanism of immune evasion. PLoS ONE.

[28] Song H., Kim J., Cosman D., Choi I. (2006). Soluble ULBP suppresses natural killer cell activity via down-regulating NKG2D expression. Cell Immunol..

[29] Coudert J.D., Scarpellino L., Gros F., Vivier E., Held W. (2008). Sustained NKG2D engagement induces cross-tolerance of multiple distinct NK cell activation pathways. Blood.

[30] Koch C., Kim Y., Zöller T., Born C., Steinle A. (2017). Chronic NKG2D Engagement In Vivo Differentially Impacts NK Cell Responsiveness by Activating NK Receptors. Front. Immunol..

[31] Barrow A.D., Cella M., Edeling M.A., Khan A.-A., Cervantes-Barragan L., Bugatti M., Schmedt C., Vermi W., Colonna M. (2024). Cutting Edge: PDGF-DD Binding to NKp44 Costimulates TLR9 Signaling and Proinflammatory Cytokine Secretion in Human Plasmacytoid Dendritic Cells. J. Immunol..

[32] Barrow A.D., Edeling M.A., Trifonov V., Luo J., Goyal P., Bohl B., Bando J.K., Kim A.H., Walker J., Andahazy M. (2018). Natural Killer Cells Control Tumor Growth by Sensing a Growth Factor. Cell.

[33] Salih H.R., Antropius H., Gieseke F., Lutz S.Z., Kanz L., Rammensee H.-G., Steinle A. (2003). Functional expression and release of ligands for the activating immunoreceptor NKG2D in leukemia. Blood.

[34] Hilpert J., Grosse-Hovest L., Grünebach F., Buechele C., Nuebling T., Raum T., Steinle A., Salih H.R. (2012). Comprehensive analysis of NKG2D ligand expression and release in leukemia: Implications for NKG2D-mediated NK cell responses. J. Immunol..

[35] Champsaur M., Lanier L.L. (2010). Effect of NKG2D ligand expression on host immune responses. Immunol. Rev..

[36] Guerra N., Tan Y.X., Joncker N.T., Choy A., Gallardo F., Xiong N., Knoblaugh S., Cado D., Greenberg N.R., Raulet D.H. (2008). NKG2D-deficient mice are defective in tumor surveillance in models of spontaneous malignancy. Immunity.

[37] Maccalli C., Giannarelli D., Chiarucci C., Cutaia O., Giacobini G., Hendrickx W., Amato G., Annesi D., Bedognetti D., Altomonte M. (2017). Soluble NKG2D ligands are biomarkers associated with the clinical outcome to immune checkpoint blockade therapy of metastatic melanoma patients. Oncoimmunology.

[38] Märten A., von Lilienfeld-Toal M., Büchler M.W., Schmidt J. (2006). Soluble MIC is elevated in the serum of patients with pancreatic carcinoma diminishing gammadelta T cell cytotoxicity. Int. J. Cancer.

[39] McGilvray R.W., Eagle R.A., Rolland P., Jafferji I., Trowsdale J., Durrant L.G. (2010). ULBP2 and RAET1E NKG2D ligands are independent predictors of poor prognosis in ovarian cancer patients. Int. J. Cancer.

[40] Xu H., Wang G., Zhu L., Liu H., Li B. (2020). Eight immune-related genes predict survival outcomes and immune characteristics in breast cancer. Aging.

[41] Zhang J., Basher F., Wu J.D. (2015). NKG2D Ligands in Tumor Immunity: Two Sides of a Coin. Front. Immunol..

[42] Waldhauer I., Steinle A. (2006). Proteolytic release of soluble UL16-binding protein 2 from tumor cells. Cancer Res..

[43] Roshani R., Boroujerdnia M.G., Talaiezadeh A.H., Khodadadi A. (2016). Assessment of changes in expression and presentation of NKG2D under influence of MICA serum factor in different stages of breast cancer. Tumor Biol..

[44] Zhao Y., Chen N., Yu Y., Zhou L., Niu C., Liu Y., Tian H., Lv Z., Han F., Cui J. (2017). Prognostic value of MICA/B in cancers: A systematic review and meta-analysis. Oncotarget.

[45] Shen J., Pan J., Du C., Si W., Yao M., Xu L., Zheng H., Xu M., Chen D., Wang S. (2017). Silencing NKG2D ligand-targeting miRNAs enhances natural killer cell-mediated cytotoxicity in breast cancer. Cell Death Dis..

[46] Liu H., Wang S., Xin J., Wang J., Yao C., Zhang Z. (2019). Role of NKG2D and its ligands in cancer immunotherapy. Am. J. Cancer Res..

[47] Shiraishi K., Mimura K., Kua L.-F., Koh V., Siang L.K., Nakajima S., Fujii H., Shabbir A., Yong W.-P., So J. (2016). Inhibition of MMP activity can restore NKG2D ligand expression in gastric cancer, leading to improved NK cell susceptibility. J. Gastroenterol..

[48] Zocchi M.R., Camodeca C., Nuti E., Rossello A., Venè R., Tosetti F., Dapino I., Costa D., Musso A., Poggi A. (2016). ADAM10 new selective inhibitors reduce NKG2D ligand release sensitizing Hodgkin lymphoma cells to NKG2D-mediated killing. Oncoimmunology.

[49] Kaiser B.K., Yim D., Chow I.-T., Gonzalez S., Dai Z., Mann H.H., Strong R.K., Groh V., Spies T. (2007). Disulphide-isomerase-enabled shedding of tumour-associated NKG2D ligands. Nature.

[50] Ferrari de Andrade L., Tay R.E., Pan D., Luoma A.M., Ito Y., Badrinath S., Tsoucas D., Franz B., May K.F., Harvey C.J. (2018). Antibody-mediated inhibition of MICA and MICB shedding promotes NK cell-driven tumor immunity. Science.

[51] Wu J. (2016). Antibody targeting soluble NKG2D ligand sMIC refuels and invigorates the endogenous immune system to fight cancer. OncoImmunology.

[52] Whalen K.A., Rakhra K., Mehta N.K., Steinle A., Michaelson J.S., Baeuerle P.A. (2023). Engaging natural killer cells for cancer therapy via NKG2D, CD16A and other receptors. MAbs.

[53] Paczulla A.M., Rothfelder K., Raffel S., Konantz M., Steinbacher J., Wang H., Tandler C., Mbarga M., Schaefer T., Falcone M. (2019). Absence of NKG2D ligands defines leukaemia stem cells and mediates their immune evasion. Nature.

[54] Gil Del Alcazar C.R., Trinh A., Alečković M., Jimenez E.R., Harper N.W., Oliphant M.U., Xie S., Krop E.D., Lulseged B., Murphy K.C. (2022). Insights into Immune Escape during Tumor Evolution and Response to Immunotherapy Using a Rat Model of Breast Cancer. Cancer Immunol. Res..

[55] Arjmandi F.K., Dogan B.E., Pinker K., Mann R., Partridge S. (2022). Chapter 13—Neoadjuvant Therapy Response Assessment with Breast MRI, in Advances in Magnetic Resonance Technology and Applications.

[56] Dang X., Zhang X., Gao Y., Song H. (2022). Assessment of Neoadjuvant Treatment Response Using Automated Breast Ultrasound in Breast Cancer. J. Breast Cancer.

[57] Janssen L.M., Dekker B.M.D., Gilhuijs K.G.A., van Diest P.J., van der Wall E., Elias S.G. (2022). MRI to assess response after neoadjuvant chemotherapy in breast cancer subtypes: A systematic review and meta-analysis. NPJ Breast Cancer.

[58] Rodenhuis S., Mandjes I.A.M., Wesseling J., van de Vijver M.J., Peeters M.-J.T.D.F.V., Sonke G.S., Linn S.C. (2010). A simple system for grading the response of breast cancer to neoadjuvant chemotherapy. Ann. Oncol..

[59] Romeo V., Accardo G., Perillo T., Basso L., Garbino N., Nicolai E., Maurea S., Salvatore M. (2021). Assessment and Prediction of Response to Neoadjuvant Chemotherapy in Breast Cancer: A Comparison of Imaging Modalities and Future Perspectives. Cancers.

[60] Kaidun P., Holzmayer S.J., Greiner S.M., Seller A., Tegeler C.M., Hagelstein I., Mauermann J., Engler T., Koch A., Hartkopf A.D. (2023). Targeting NKG2DL with Bispecific NKG2D-CD16 and NKG2D-CD3 Fusion Proteins on Triple-Negative Breast Cancer. Int. J. Mol. Sci..

[61] Therneau T.M., Lumley T. (2015). Package ‘survival’. R. Top. Doc..

[62] Kassambara A., Kosinski M., Biecek P., Fabian S. (2017). “Package ‘Survminer’.” Drawing Survival Curves Using ‘ggplot2’ (R Package Version 03 1) 3. https://cran.r-project.org/web/packages/survminer/survminer.pdf.

