# Supplementary material for: Soluble NKG2DLs Are Elevated in Breast Cancer Patients and Associate with Disease Outcome
Source: Int J Mol Sci. 2024 Apr 8;25(7):4126. doi: 10.3390/ijms25074126 (PMC11012452; doi:10.3390/ijms25074126)
Supplement: Supplementary file 1 [file ijms-25-04126-s001.zip › ijms-2931831-supplementary.pdf]

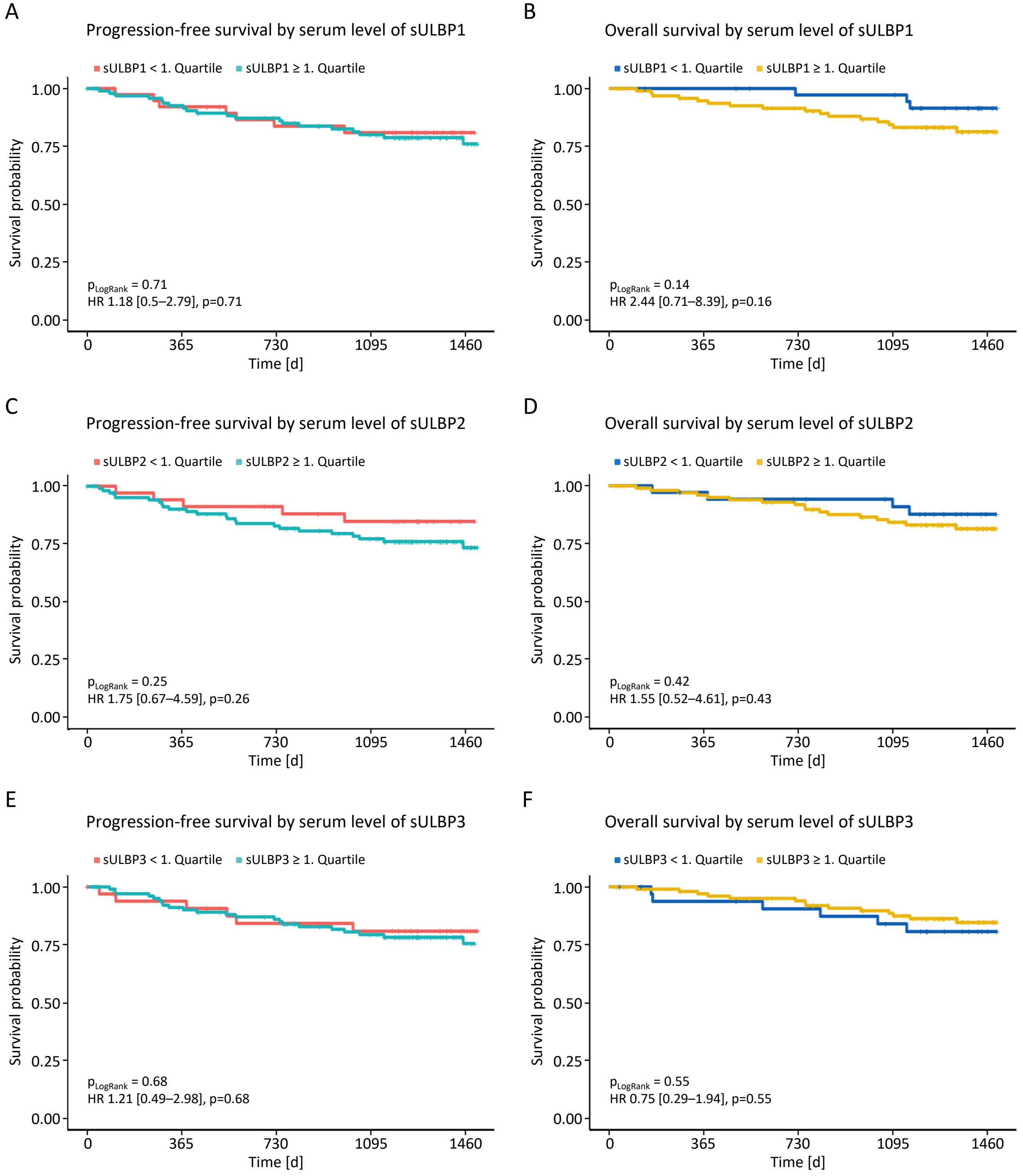

Supplemental Figure S1. Correlation between sNKG2DL serum levels and PFS and OS in BC patients.

(**A–F**) Serum levels of sNKG2DL were determined through the use of ELISA in BC patients (n = 140) and the correlation between sULBP1 (**A**,**B**), sULBP2 (**C**,**D**), and sULBP3 (**E**,**F**) and PFS and OS in BC patients below and above the first quartile is shown. PFS, progression-free survival; OS, overall survival; %; percent; *p*, *p*-value.

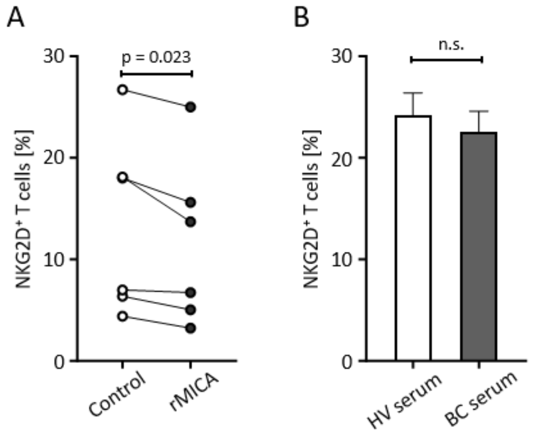

Supplemental Figure S2. sNLG2DLs induce the downregulation of NKG2D on T cells.

(**A**) PBMCs from HVs (n = 5) were co-cultured with soluble rMICA for 48 h and NKG2D expression on T cells was assessed through the use of flow cytometry. (**B**) PBMCs from HVs (n = 6) were co-cultured with serum from HVs (n = 9) or BC patients with high sNKG2DL levels (n = 6) for 24 h and NKG2D expression on T cells was assessed through the use of flow cytometry. HVs, healthy volunteers; BC, breast cancer; %; percent; *p*, *p*-value; n.s., not significant.
